# Sarcopenia is a risk factor for postoperative delirium in geriatric hip fracture patients: a retrospective study

**DOI:** 10.3389/fmed.2024.1526240

**Published:** 2025-01-06

**Authors:** Yi-Ming Qi, Hao-Tao Li, Shi-Min Chang, Sun-Jun Hu, Shou-Chao Du, Chen-Dong Liu, Yong-Qian Chu, Yun-Feng Rui

**Affiliations:** ^1^Department of Orthopaedic Surgery, Yangpu Hospital, Tongji University School of Medicine, Shanghai, China; ^2^Department of Orthopaedics, Zhongda Hospital, School of Medicine, Southeast University, Nanjing, China; ^3^Orthopaedic Trauma Institute (OTI), School of Medicine, Southeast University, Nanjing, China

**Keywords:** sarcopenia, postoperative delirium, risk factor, geriatric, hip fracture

## Abstract

**Background:**

Various factors contribute to postoperative delirium (POD) in elderly patients undergoing hip fracture surgeries. Sarcopenia was defined as the progressive loss of skeletal muscle mass and strength associated with aging. The aim of this study was to explore the prevalence of POD and sarcopenia in geriatric patients undergoing hip fracture surgeries and to investigate the correlation between preoperative sarcopenia and POD.

**Methods:**

After applying specific inclusion and exclusion criteria, the information of 234 patients were retrospectively collected. POD was screened for with 4A’s Test and diagnosed with DSM-5 criteria. The incidences of POD and sarcopenia were calculated. The demographic and perioperative features as well as comorbidities of delirious and non-delirious patients were analyzed and the risk factors analysis for POD in elderly hip fracture patients were conducted through univariate analysis and multivariate regression analysis.

**Results:**

48.7% patients were diagnosed of POD, 78.95% of which were females. The average age of delirious and non-delirious patients were 84.75 years and 80.63 years, respectively. The incidence of sarcopenia was 41.02% for all the included patients with 60.53% for delirious patients and 20.55% for non-delirious patients. Results of univariate analysis showed that sarcopenia (OR:5.281, 95%CI 2.988–9.337, *p* = 0.000), age increase per year (OR:1.128, 95CI 1.070–1.190, *p* = 0.000), operation duration increase (OR:1.017, 95%CI 1.004–1.030, *p* = 0.011), intertrochanteric fracture (OR:2.571, 95%CI 1.517–4.358, *p* = 0.000), dementia (OR: 6.029, 95%CI 2.532–14.359, *p* = 0.000), ASA > 2 (OR: 6.955, 95%CI 3.461–13.976, *p* = 0.000), coronary heart disease (OR: 2.201, 95%CI 1.257–3.854, *p* = 0.006), renal insufficiency (OR: 2.215, 95%CI 1.187–4.133, *p* = 0.012) and COPD (OR: 2.554, 95%CI 1.414–4.615, *p* = 0.002) were risk factors for POD. Results of multivariate analysis identified sarcopenia (OR: 2.457, 95% CI 1.226–4.923, *p* = 0.011), ASA > 2 (OR: 3.968, 95% CI 1.805–8.722, *p* = 0.001), dementia (OR: 3.912, 95% CI 1.390–11.014, *p* = 0.010) and coronary heart disease (OR: 2.176, 95% CI 1.044–4.538, *p* = 0.038) as independent risk factors for POD in geriatric hip fracture patients.

**Conclusion:**

The incidences of POD and sarcopenia in geriatric hip fracture patients are high. Sarcopenia is an independent risk factor for POD in geriatric hip fracture patients.

## 1 Introduction

Delirium is a reversible cognitive disorder caused by temporary damage to neurons due to an underlying systemic disturbance (1). Delirium diagnosis is elucidated in the Diagnostic and Statistical Manual of Mental Disorders, Fifth Edition (DSM-5), which is published by the American Psychiatric Association. The DSM-5 offers guidelines for classifying and diagnosing mental health disorders. As per the DSM-5, delirium is identified by acute impairments in attention, cognition, and/or consciousness that manifest rapidly, fluctuate in intensity, and are accompanied by a deviation from normal cognitive functioning. Delirium can be provoked by various potential factors, which encompass acute medical conditions, substance use or withdrawal, as well as trauma or surgical procedures (2).

Postoperative delirium (POD) is a common complication observed among patients undergoing hip surgery. The incidence of POD in hip fracture patients may reach as high as around 50% (3, 4). POD is linked to several negative outcomes, including prolonged hospital stays (5, 6), greater healthcare costs (6); diminished rehabilitation progress with impaired functional and cognitive recovery, and even the potential development of new-onset dementia (7–9) and increased short-term and long-term mortality (10, 11).

Various factors contribute to the likelihood of POD in elderly patients undergoing hip fracture surgeries. Previous research has identified advanced age, preoperative dementia, low albumin levels, diabetes, surgical delay and pain as contributors to delirium (12–14). Fortunately, delirium prevention strategies works in reducing the incidence of delirium and associated adverse outcomes (15). Therefore, it is crucial to screen for risk factors associated with POD in hip surgery patients to implement preventive measures effectively.

Sarcopenia was defined as the progressive loss of skeletal muscle mass and strength associated with aging (16). It is a common condition in older patients with hip fracture, ranging from 21 to 74% in men and 12 to 68% in women, depending on the diagnostic criteria used. In recent years, evidences globally suggested that sarcopenia is associated with cognitive impairment (17). Experimental studies indicated that delirium and sarcopenia might share similar pathophysiologic mechanisms including inflammatory reaction and reactive oxidative stress and a potential biological cross-talk (18).

However, whether sarcopenia at admission is a risk factor for POD in hip fracture patients has not been studied. Thus, we designed a study to provide insights into the issue. This study retrospectively analyzes medical records to delineate the incidence of POD and sarcopenia in geriatric hip fracture patients and explore whether sarcopenia at admission serves as a potential risk factor for POD.

## 2 Materials and methods

### 2.1 Study design and patients

After having got approval from the institutional ethical committee, this retrospective cohort study utilized data from electronic medical records at a tertiary care university hospital in Shanghai, China. From February 1, 2022 to January 31, 2024, a total of 361 patients were admitted to the tertiary hospital for hip fracture treatment.

Researchers gathered data by scrutinizing patients’ records obtained from the electronic medical records system. Inclusion criteria were defined as follows: (1) patients aged 65 years or older; (2) patients who received internal fixation for an intertrochanteric fracture or hemiarthroplasty for a femoral neck fracture; (3) hip fractures caused by low energy. Exclusion criteria were defined as follows: (1) individuals with multiple fractures including hip fractures; (2) patients demonstrating delirium at admission or before surgery; (3) patients whose fracture happened more than 3 days before admission; (4) pathological fracture; (5) the American Society of Anesthesiologists (ASA) rating scale was Classes V; (6) patients who died before surgery or with 3 days after surgery; (7) patients with incomplete medical records.

**Figure 1:**
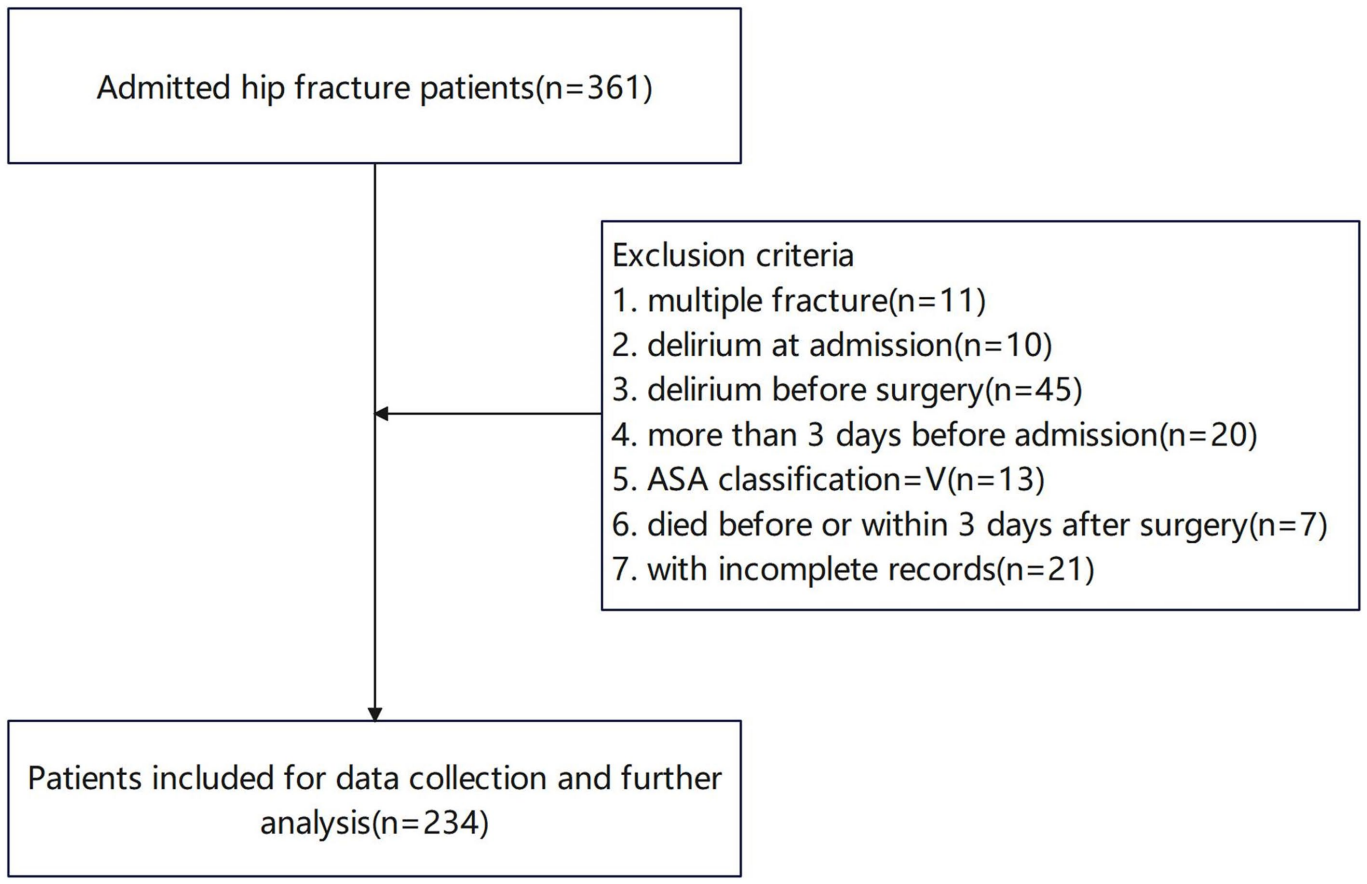
The flowchart of patient enrollment.

After applying specific inclusion and exclusion criteria, 234 patients fulfilled the criteria and their information were collected for further analysis (Figure 1).

### 2.2 Data collection

The demographic, clinical, and perioperative information of patients was obtained from the hospital information system and patients’ medical records. The first two authors reviewed the medical records to verify patients’ demographic, clinical, and perioperative information independently. Any inconsistencies or disagreements were resolved by the corresponding author.

Variables were grouped into the following categories:(1) sociodemographic data (age and gender) and body mass index (BMI); (2) past medical history (dementia, sarcopenia, hypertension, coronary heart disease, diabetes mellitus, chronic obstructive pulmonary diseases (COPD), stroke, and renal insufficiency); (3) post-injury factors (operation delay, fracture type), among which the operation delay means the days from admission to operation; (4) surgery related factors [ASA classification, anesthesia type, operation duration (min), and whether the patient received intraoperative blood transfusion] and post-operative factors (the lowest hemoglobin within 5 postoperative days, the lowest albumin within 5 postoperative days, and whether the patient received transfusion within 5 days after surgery).

### 2.3 Assessment criteria for delirium and sarcopenia

Delirium was screened with the 4AT (19). The 4AT is a brief and pragmatic tool for the screening of delirium which has demonstrated acceptable diagnostic test accuracy in acute medical wards. A score higher than 4 is strongly predictive of delirium. Patients screened positive during the 4AT assessment will be further evaluated for the presence of delirium using the DSM-5 criteria (20). The sarcopenia was evaluated for every patient on the day of admission. Sarcopenia was evaluated according to the Asian Working Group for Sarcopenia (AWGS) 2019 consensus. Those who demonstrated both low muscle strength (handgrip strength <28 kg for men and <18 kg for women) and low calf circumference (<34 cm in men, <33 cm in women) were identified as sarcopenia patients (46).

### 2.4 Statistical analysis

We conducted statistical analysis with SPSS 23.0 software. Statistical significance was established at a threshold of *p* < 0.05. Measurement data are depicted as mean ± standard deviation (mean ± SD). To compare data between two groups, we employed the *t*-test. Counting data underwent analysis using the chi-squared test (*χ*^2^). The Variance Inflation Factor (VIF) was employed to assess multicollinearity among the variables and measure the extent to which the estimated regression coefficient is inflated because of correlations among predictor variables. A cut-off point was established, with the VIF being required to be less than 5. We found that the VIFs of all the variables were less than 5. Following this, the Hosmer–Lemeshow Test was conducted to assess the goodness of fit of the binary logistic regression model. We got a *p*-value of 0.144 that was larger than 0.05, which showed that the model fits well. Then we conducted a comparison between patients with delirium and those without using univariate analysis. Variables with a *p*-value less than 0.05 from the univariate analysis were included in the multivariate analysis to ascertain the independent risk factors for POD. The Graphpad Prism 10.1.2 was use to generalize the forest plot for the visualization of multivariate logistic regression analysis results.

## 3 Results

### 3.1 Subjects’ characteristics

A flowchart depicting the screening process for hip fracture patients derived from our center’s hip fracture database was presented in Figure 1. After excluding patients who did not meet the inclusion criteria, a total of 234 patients were deemed eligible for the study.

The demographic and perioperative characteristics of these 234 patients were showed in Table 1. 48.72% patients were diagnosed of POD, 78.95% of which were females. The delirious patients were significantly older than non-delirious patients (84.75 vs. 80.63 years, *p* = 0.000). In delirious patients, 63.16% patients suffered from intertrochanteric fractures while only 40% non-delirious patients were diagnosed with intertrochanteric fractures (*p* = 0.000). The incidence of sarcopenia was 41.02% for all the included patients and the incidence in delirious patients was about 3 times (60.53% vs. 20.55%, *p* = 0.000) that in non-delirious patients. The average waiting time for surgery from admission to surgery were 4.37 days and 3.95 days, respectively, for the two group (*p* = 0.128). In the two groups, 89.47% (102 out of 114) and 55.00% (66 out of 120) patients had an ASA level > 2 (*p* = 0.000). Two hundred sixteen patients received spinal anesthesia and 78 patients received intraoperative/postoperative blood transfusion (*p* = 0.113). Besides, delirious patients showed significantly larger incidence of dementia (27.19% vs. 5.83%, *p* = 0.000), coronary heart disease (41.23% vs. 24.17%, *p* = 0.005), renal insufficiency (30.70% vs. 16.67%, *p* = 0.011), COPD (37.72% vs. 19.17%, *p* = 0.002), longer operation duration (81.32 ± 21.11 min vs. 74.45 ± 19.33 min, *p* = 0.010), lower postoperative hemoglobin level (91.11 ± 15.89 g/L vs. 95.85 ± 17.04 g/L, *p* = 0.029) and albumin level (29.90 ± 2.51 g/L vs. 31.02 ± 2.85 g/L, *p* = 0.002).

**Table 1:** Demographic and perioperative features of delirious and non-delirious patients.

Variables	Delirium (114)	Non-delirium (120)	*p-*value
Age	84.75 ± 4.94	80.63 ± 8.344	0.000
Gender			0.474
Female, *n*(%)	90(78.95%)	90(75.00%)	
Male, *n*(%)	24(21.05%)	30(25.00%)	
BMI, mean ± SD, kg/m^2^	21.68 ± 2.57	21.91 ± 2.35	0.471
Fracture type, *n*(%)			0.000
Femoral neck fracture	42(36.84%)	72(60.00%)	
Intertrochanteric fracture	72(63.16%)	48(40.00%)	
Sarcopenia, *n*(%)	69(60.53%)	27(22.50%)	0.000
Dementia, *n*(%)	31(27.19%)	7(5.83%)	0.000
Diabetes mellitus, *n*(%)	63(55.26%)	57(47.50%)	0.235
Hypertension, *n*(%)	78(68.42%)	69(57.50%)	0.084
Coronary heart disease, *n*(%)	47(41.23%)	29(24.17%)	0.005
Renal insufficiency, *n*(%)	35(30.70%)	20(16.67%)	0.011
Stroke, *n*(%)	30(26.32%)	24(20.00%)	0.252
COPD, *n*(%)	43(37.72%)	23(19.17%)	0.002
ASA classification, *n*(%)			0.000
ASA level > 2	102(89.47%)	66(55.00%)	
ASA ≤ 2	12(10.53%)	54(45.00%)	
Operation delay, mean ± SD, day	4.37 ± 2.29	3.95 ± 1.89	0.128
Operation duration, mean ± SD, min	81.32 ± 21.11	74.45 ± 19.33	0.010
Anesthesia type, *n*(%)			0.113
Spinal anesthesia	102(89.47%)	114(95.00%)	
General anesthesia	12(10.53%)	6(5.00%)	
Blood transfusion, *n*(%)	42(36.84%)	36(30.00%)	0.267
Postoperative Hb, mean ± SD, g/L	91.11 ± 15.89	95.85 ± 17.04	0.029
Postoperative ALB, mean ± SD, g/L	29.90 ± 2.51	31.02 ± 2.85	0.002

### 3.2 The results of univariate analysis

Risk factors for POD in geriatric hip fracture patients were analyzed. The results of univariate analysis of risk factors for POD in elderly hip fracture patients showed that patients with sarcopenia were at a higher risk of POD (OR: 5.281, 95% CI 2.988–9.337, *p* = 0.000). Further, age increase (OR: 1.128, 95% CI 1.070–1.190, p = 0.000), operation duration increase (OR: 1.017, 95% CI 1.004–1.030, *p* = 0.011) also increased the incidence of delirium. Intertrochanteric fracture patients suffered more from POD compared with femoral neck fracture patients (OR: 2.571, 95% CI 1.517–4.358, *p* = 0.000). Those with coexisting dementia were also more likely to suffer from POD than those without preoperative dementia (OR: 6.029, 95% CI 2.532–14.359, *p* = 0.000). Besides, ASA > 2, coronary heart disease, renal insufficiency and COPD were risk factors for POD (Table 2).

**Table 2:** Univariate analysis of risk factors for POD in elderly hip fracture patients.

Variables	OR (95% CI)	*p*-value
Age, per year	1.128(1.070–1.190)	0.000
Gender, female	0.800(0.434–1.474)	0.474
BMI	0.962(0.866–1.068)	0.469
Fracture type (intertrochanteric fracture)	2.571(1.517–4.358)	0.000
Sarcopenia	5.281(2.988–9.337)	0.000
Dementia	6.029 (2.532–14.359)	0.000
Diabetes mellitus	1.365(0.816–2.284)	0.235
Hypertension	1.601(0.937–2.736)	0.085
Coronary heart disease	2.201(1.257–3.854)	0.006
Renal insufficiency	2.215(1.187–4.133)	0.012
Stroke	1.429(0.775–2.633)	0.253
COPD	2.554(1.414–4.615)	0.002
ASA > 2	6.955(3.461–13.976)	0.000
Operation delay, per day	1.101(0.972–1.248)	0.130
Operation duration, per min	1.017(1.004–1.030)	0.011
Anesthesia type (General anesthesia)	2.235(0.809–6.173)	0.121
Blood transfusion	1.361(0.789–2.348)	0.268
Postoperative Hb > 90 g/L	0.740(0.437–1.256)	0.265
Postoperative ALB>30 g/L	0.741(0.441–1.244)	0.256

### 3.3 The results of multivariate logistic regression analysis

**Figure 2:**
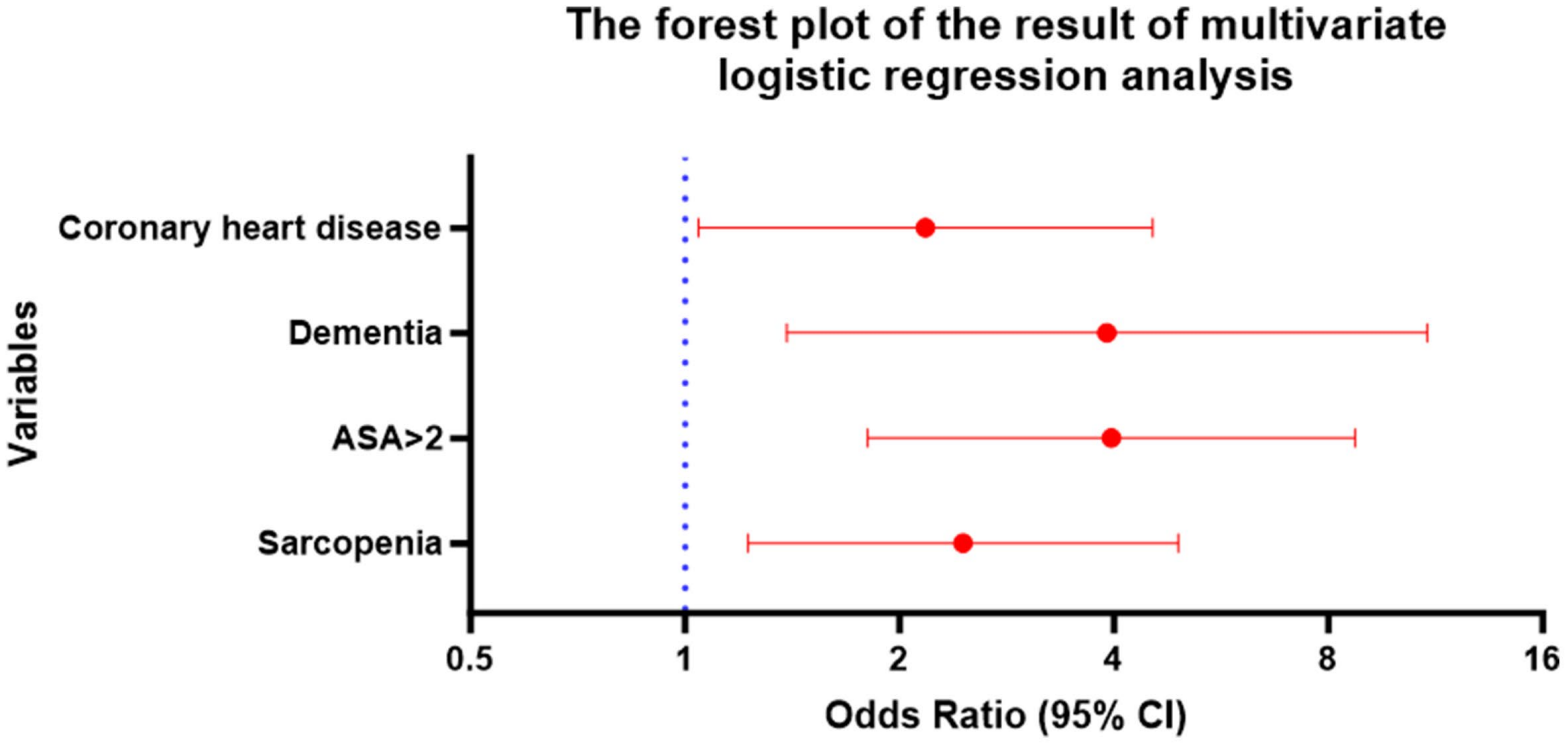
The forest plot of the result of multivariate logistic regression analysis.

Multivariate logistic regression analysis was performed using variables showing statistical significance in the univariate analysis. Results of multivariate analysis identified sarcopenia (OR: 2.457, 95% CI 1.226–4.923, *p* = 0.011), ASA > 2 (OR: 3.968, 95% CI 1.805–8.722, *p* = 0.001), dementia (OR: 3.912, 95% CI 1.390–11.014, *p* = 0.010) and coronary heart disease (OR: 2.176, 95% CI 1.044–4.538, *p* = 0.038) as independent risk factors for POD in geriatric hip fracture patients (Table 3 and Figure 2).

**Table 3:** Multivariate logistic regression analysis of risk factors for POD.

Variables	OR (95% CI)	*P-*value
Sarcopenia	2.457(1.226–4.923)	0.011
ASA > 2	3.968(1.805–8.722)	0.001
Dementia	3.912(1.390–11.014)	0.010
Coronary heart disease	2.176(1.044–4.538)	0.038

## 4 Discussion

The POD is a common postoperative complication in geriatric hip fracture patients. Its high incidence and adverse outcomes make it crucial to identify modifiable risk factors and to implement effective interventions to prevent its occurrence (1). Despite loads of studies investigated risk factors for POD, to our knowledge, this is the first study to explore the potential association between sarcopenia and POD in hip fracture patients. The results provided a compelling evidence of a link between sarcopenia and POD in older patients undergoing hip fracture surgeries. Consequently, clinicians should be vigilant for postoperative delirium in patients who are diagnosed with preoperative sarcopenia. Then actions including analgesics, multi-disciplinary comprehensive care and pharmacological treatment should be taken for postoperative delirium prevention (10).

### 4.1 High incidence of POD and sarcopenia

We identified the high incidences of POD (48.72%) and sarcopenia (41.02%), which are compatible to some former studies. In our study, we excluded 55 delirious patients at admission or before surgery, so the incidence of POD in the whole patients may be higher than this. In the studies of Jeon and Sohng et al. (22) and Shin et al. (4), 45.02 and 51.28% patients were diagnosed of delirium, respectively. In the study of Oberai et al. (23) in 2021 including 6,672 geriatric hip fracture patients, 2,599 (39.0%) patients were delirious. The incidence of sarcopenia in hip fracture patients may reach about 50% in different races (21, 24, 25). In our study, the incidence of sarcopenia was less than 50% (41.02%) for all the included patients. This may due to that patients with preoperative delirium or delirium at admission were excluded, who may be more susceptible to sarcopenia. According to former studies, sarcopenia is a risk factor for hip fractures (26–28), which underscores the importance of addressing possible sarcopenia as a preventive measure to reduce the incidence of hip fractures. The high incidence may result from that sarcopenia is associated with osteoporosis and falls among elderly people (29). And the coexistence of osteoporosis and sarcopenia has been recently defined as a syndrome termed ‘osteosarcopenia’. Besides, the prevalence of sarcopenia will increase after hip fracture surgeries (27). Postoperative treatment concerning sarcopenia including long-term exercise therapy and anti-osteoporosis drugs should be utilized to decrease the incidence of postoperative sarcopenia and to prevent the aggravation of sarcopenia (27).

### 4.2 Clinical studies concerning the association between POD and sarcopenia

Very few papers studied the association between delirium and sarcopenia. Mosk et al. (30) found that low skeletal muscle mass was independently associated with POD in elderly patients undergoing colorectal cancer surgery. Zucchelli et al. (31) verified that low muscle mass is independently associated with delirium in acute hospital medical wards, emergency departments, rehabilitation wards, nursing homes and hospices in Italy. However, these studies only explored the association between POD and low muscle mass rather than sarcopenia. According to Dong et al. (32), preoperative sarcopenia was independently associated with POD in geriatric patients after gastrointestinal cancer surgery. Moellmann et al. (33) carried out a prospective clinical study on 421 patients from 4 different surgical disciplines including orthopedic and trauma, finding that if there is an indication of sarcopenia, the association with delirium will increase 5.56-fold. Our study is the first study having identified sarcopenia as an independent risk factor for POD in elderly hip fractures.

### 4.3 Potential pathophysiological mechanisms shared by POD and sarcopenia

Although little studies have been conducted to explore the shared pathophysiological mechanisms linking brain and muscle disorders, there are still some evidences suggesting similar pathophysiologic mechanisms that may be shared by delirium and sarcopenia.

Firstly, elderly hip fracture patients often show elevated serum levels of pro-inflammatory cytokines, including tumor necrosis factor-alpha (TNF-*α*), interleukin-1 (IL-1), IL-6, and IL-8 (18, 34). Once these inflammatory mediators cross the blood–brain barrier, they can activate microglia, resulting in synaptic and neuronal dysfunction, which may ultimately contribute to the onset of delirium (35). Meanwhile, inflammatory reaction plays an important role in sarcopenia development. Elevated IL-6 and TNF-α plasma level were found in geriatric patients and TNF-α was proved to be a factor associated with muscle mass and strength decline (36–38). Secondly, reactive oxidative stress (ROS) was found to be associated with both delirium and sarcopenia. Under specific circumstances, such as during surgery, the brain becomes particularly susceptible to damage from oxidative stress, which may result in neuronal dysfunction and delirium (39). ROS was found directly associated with muscle mass in normal aging and the intake of antioxidants, circulating antioxidant levels, and markers of oxidative damage have shown varying correlations with sarcopenia (40, 41). Thirdly, the direct association between them was provided by Nemoto et al. (42) that low skeletal muscle mass is associated with perioperative neurocognitive disorder due to decreased neurogenesis.

### 4.4 Other risk factors for POD

In our study, higher ASA level was identified as an independent risk factor for POD. This is consistent with some previous research findings of Haynes et al. (43), Kim et al. (44), and Wang et al. (14). It suggested that physiological reserve may be associated with the evolution of cognitive deficits after surgery for the preoperative ASA classification serves as an initial evaluation of a patient’s ability to tolerate anesthesia and provides a fundamental indication of their overall physical condition.

As in our study, many former studies recognized dementia as an independent risk factor for POD (13, 43, 44). As two distinct clinical syndromes, delirium and dementia are connected by overlapping clinical characteristics and shared pathogenic mechanisms. Delirium can influence the progression of dementia by accelerating cognitive decline, functional deterioration, and the loss of independence. Furthermore, delirium may serve as an initial indicator of dementia (34).

Seldom studies found that coronary heart disease act as a risk factor for POD. Wang et al. found that delirium was significantly associated with coronary heart disease through univariate analysis (45). However, it was found an insignificant factor through multivariate regression analysis. Our study suggests that more attention be paid to those suffering from coronary heart disease.

Some former studies listed age increase and male as independent risk factors for POD (43–45), although the results of our multivariate logistic regression analysis show that advanced age and male are not an independent risk factor. More attention still should be paid to older patients in routine care.

### 4.5 Strengths and limitations

The findings of our study should be interpreted considering certain limitations. Firstly, this is a single-center retrospective study, and the sample size is not very big. In the future, multi-center prospective cohort studies of large samples should be conducted for further verification. Secondly, the diagnosis of sarcopenia were based on low muscle strength and low calf circumference. Although calf circumference is strongly correlated with skeletal muscle mass, patients with obesity or other conditions that can lead to increased calf circumference and decreased muscle mass may not have been accurately identified. Lastly, as those diagnosed with delirium at admission or before operation were excluded, our findings need further validation for whether the results can be generalized to all the hip fracture patients.

Strength of this study is that it is the first study to list sarcopenia as a potential risk factor for POD and has identified sarcopenia as an independent risk factor for POD in elderly hip fractures. This provides a reference for the identification of high-risk patients for postoperative delirium. Besides, delirium was screened with validated screening tools and diagnosed with standard criteria during routine care.

## 5 Conclusion

In conclusion, the incidences of POD and sarcopenia in hip fracture patients are high. Sarcopenia is an independent risk factor for POD in geriatric hip fracture patients. We recommend prospective large-sample clinical studies and experimental studies exploring the underlying mechanisms of the association between POD and sarcopenia be conducted in the future.

## Data Availability

The raw data supporting the conclusions of this article will be made available by the authors, without undue reservation.
